# Effects of a thrombomodulin-derived peptide on monocyte adhesion and intercellular adhesion molecule-1 expression in lipopolysaccharide-induced endothelial cells

**Published:** 2013-02-01

**Authors:** Yan Xu, Xun Xu, Huiyi Jin, Xiaolu Yang, Qing Gu, Kun Liu

**Affiliations:** Shanghai Key Laboratory of Ocular Fundus Diseases, Department of Ophthalmology, Shanghai First People’s Hospital Affiliated to Shanghai Jiaotong University, Shanghai, PR China

## Abstract

**Purpose:**

It has been documented that GC31, a 31-animo acid peptide from human thrombomodulin, has potent anti-inflammatory properties in endotoxin-induced uveitis and lipopolysaccharide (LPS)-induced RAW264.7 cells, while the role of GC31 in the endothelial cells has not yet been fully understood. Therefore, the aim of this study was to explore the effect of GC31 on intercellular adhesion molecule-1 (ICAM-1) expression in LPS-activated endothelial cells.

**Methods:**

Human umbilical vein endothelial cells (HUVECs) were incubated with LPS (1 μg/ml) and peptide GC31 or control peptide VP30 simultaneously. ICAM-1 messenger RNA and protein levels were evaluated with real-time PCR and western blot. The adhesion of U937 cells labeled with CM-H2DCFDA to HUVECs was examined with ﬂuorescence microscope. Extracellular signal-regulated kinase-1/2 (ERK1/2) and p38 mitogen-activated protein kinase (MAPK) activation, inhibitor of nuclear factor kappa B alpha (IκBα) degradation, and nuclear factor kappa B (NF-κB) nuclear translocation were detected with western blot.

**Results:**

Upon LPS stimulation, GC31 suppressed the mRNA and protein expression of ICAM-1 in HUVECs and remarkably reduced monocyte-endothelial cell adhesion in a dose-dependent manner. Furthermore, GC31 significantly inhibited the degradation of IκBα and nuclear translocation of NF-κB and moderately blocked the activation of p38 MAPK and ERK1/2 in activated HUVECs.

**Conclusions:**

Our results suggested that GC31 suppressed LPS-mediated ICAM-1 expression by inhibiting the activation of NF-κB and partially by attenuating the activity of ERK1/2 and p38 MAPK in vascular endothelium, which may contribute to ameliorating vascular inflammatory diseases, such as uveitis.

## Introduction

Lipopolysaccharide (LPS), an essential component of the surface of Gram-negative bacteria [1], has potent proinflammatory properties in many cell types [2-4], including endothelial cells [5,6]. A major consequence of the LPS action on endothelial cells is the upregulation of genes specifically involved in recruiting and adhering leukocytes [7].The firm adhesion of leukocytes to the vessel wall occurs via interaction of the CD11a/CD18 (β2) integrins to endothelial ligands such as intercellular adhesion molecule-1 (ICAM-1). ICAM-1, an inducible cell transmembrane glycoprotein, acts as a key component in inﬂammatory response for recruiting leukocytes to the sites of inﬂammation and is implicated in the pathogenesis of numerous inﬂammatory diseases such as rheumatoid arthritis [8], uveitis [9], and atherosclerosis [10,11]. Thus, it is suggested that modulation of adhesion molecule expression and reduction of aberrant leukocyte adhesion to the endothelium may be an attractive approach for treating inﬂammation-related vascular complications, including inflammatory ocular disorders [12].

We previously demonstrated that the peptide GC31, which is derived from C-type lectin-like domain (CTLD) of human thrombomodulin (TM), has a potent anti-inflammatory effect on endotoxin-induced uveitis (EIU) by reducing leukocyte infiltration and proinflammatory mediator expression [13]. Uveitis is characterized by an increase in leukocyte rolling, sticking, and adhesion molecule expression, and breakdown of the blood–retinal barrier, which subsequently leads to transendothelial migration of leukocytes and recruitment of large numbers of cells to the retina [14-16]. GC31 intravitreal injection could reduce leukocyte counts in aqueous humor and leukocyte infiltration in the anterior chamber, iris-ciliary bodies, and posterior vitreous according to histological examination. In addition, it has been reported that TM CTLD dampens the inflammatory response by interfering with leukocytes adhesion through inhibiting adhesion molecule expression [17]. Despite those studies, it is still unknown whether the effect of GC31 on EIU is mediated by inhibiting leukocyte-endothelium adhesion. Thus, the aim of the present study was to investigate the ability of GC31 to modulate the expression of ICAM-1 in LPS-induced HUVECs and to identify the underlying mechanism(s).

## Methods

### Reagents

Rosewell Park Memorial Institute (RPMI) 1640, fetal bovine serum (FBS), and antibiotics were from Gibco-BRL (Grand Island, NY). LPS (*E. coli* 055:B5) was purchased from Sigma (St. Louis, MO). The polyclonal antibodies against p38 mitogen-activated protein kinase (MAPK), phospho-p38 (p-p38) MAPK, extracellular signal-regulated kinase-1/2 (ERK1/2), phospho-ERK1/2 (p-ERK1/2), inhibitor of nuclear factor kappa B alpha (IκBα), phospho-IκBα (p-IκBα), and phosphonuclear factor kappa B (NF-κB) p65 (p-NF-κB p65) were obtained from Cell Signaling Technology, Inc. (Beverly, MA). The rabbit monoclonal antibodies against ICAM-1 and Lamin A/C were obtained from Epitomics, Inc. (Burlingame, CA). Horseradish peroxidase (HRP)-conjugated monoclonal mouse antiglyceraldehyde-3-phosphate dehydrogenase (GAPDH) was from Kangchen Biotech (Shanghai, China). Goat antirabbit immunoglobulin G (Ig G) was from R&D Systems (Minneapolis, MN).

### Synthesis of peptide

Peptide GC31 and control peptide VP30 were synthesized using high-efﬁciency solid-phase peptide synthesis with an automatic peptide synthesizer (Symphony; Protein Technologies, Tucson, AZ) and performed by ChinaPeptides Co., Ltd. in Shanghai, PR China. The purity over 95% was characterized by analytical high-performance liquid chromatography and mass spectrometry. All the synthesized peptides were freeze-dried and stored at −20 °C until used. The peptides were dissolved in culture medium and sterilized by filtration through a 0.2 μm filter. All the synthesized peptides were freeze-dried and stored at −20 °C until used. The peptides were dissolved in culture medium and sterilized by filtration through a 0.2 μm filter.

### Cell culture and treatment

Human umbilical vein endothelial cells (HUVECs) were obtained from ScienCell (San Diego, CA). The cells were maintained in endothelial cell medium (ECM; ScienCell) consisting of 500 ml of basal medium, 25 ml of fetal bovine serum, 5 ml of endothelial cell growth supplement, and 5 ml of penicillin/streptomycin in a humidified incubator at 37 °C with an atmosphere of 5% CO_2_. HUVECs were used at passages 3–8 and allowed to grow to subconfluence before treatment. Before the treatment with LPS or peptides, the culture medium was replaced by fresh medium, and LPS was added at a final concentration of 1 μg/ml. GC31 (0.1–10 μM) or VP30 (10 μM) peptides were added to the culture medium simultaneously with LPS. The monocyte U937 cell lines (human monocytic leukemia cell line U937) were purchased from the Cell Bank of Shanghai Institutes for Biologic Sciences (Shanghai, China). Cells were maintained in RPMI 1640 supplemented with 10% FBS, 100 IU/ml penicillin, and 100 μg/ml streptomycin in a humidified incubator at 37 °C with an atmosphere of 5% CO_2_.

### Cell viability assay

The cell viability of the HUVECs was assessed using the CellTiter 96 aqueous one solution cell proliferation assay (MTS) kit (Promega Corporation, Madison, WI). Briefly, 2×10^4^ cells in 100 μl of medium were seeded in each well of a 96-well plate and allowed to adhere for 48 h. Then cells were treated with various concentrations of peptides or LPS (1 μg/ml) in ECM with 0.1% FBS for 24 h. Cells in the control group were left untreated. Afterwards, 20 μl of MTS solution was added in each well and incubated for another 3 h at 37 °C. Absorbance was detected at 490 nm with a microplate reader (Model 680; Bio-Rad, Hercules, CA).

### Monocytoid cell adhesion assays

The effect of GC31 on the adhesion of U937 cells to LPS-activated HUVECs was evaluated as previously described [18,19]. For the adhesion assays, HUVECs were grown to confluence in 24-well tissue culture plates, pretreated with various concentrations of GC31 or VP30 for 18 h, and then cells were treated with LPS with a final concentration of 1 μg/mL for an extra 6 h. U937 cells were suspended overnight in RPMI 1640 medium containing 0.1% FBS and freshly harvested and labeled with CM-H2DCFDA (10 µM, Invitrogen, Grand Island, NY) in 0.1% FBS RPMI 1640 medium at 37 °C for 30 min. Fluorescence-labeled U937 cells were washed three times with fresh RPMI 1640 medium, and then added at a density of 5×10^4^ cells/well onto the 24-well plate containing pretreated HUVECs. After incubation at 37 °C for 30 min, non-adhered U937 cells were removed by gentle washing with PBS (NaCl 8.0 g, KCl 0.2 g, Na_2_HPO_4_•12H_2_O 2.9 g, KH_2_PO_4_ 0.2 g, and distilled water 1 L), and the monolayers were fixed with 4% paraformaldehyde in PBS. The number of adhered U937 cells was counted in five random fields (10× objective) per well in a masked fashion and expressed as the number of adhered cells per field. The images were captured under a confocal ﬂuorescence microscope (LSM 510; Carl Zeiss, Gottingen, Germany). Experiments were performed in triplicate and repeated at least three times.

### Western blot analysis for intercellular adhesion molecule-1

Crude proteins were extracted from HUVECs treated with 1 μg/ml LPS in the presence or absence of GC31 (0.1–10 μM) or VP30 (10 μM) for 12 h. Protein concentration was determined using the Bio-Rad protein assay (Bio-Rad Lab). Proteins were then resolved on 10% sodium dodecyl sulfate–polyacrylamide gel electrophoresis (SDS–PAGE; Bio-Rad) gels. After electrophoresis, the proteins were electrotransferred to polyvinylidene ﬂuoride (PVDF) membrane (Millipore, Billerica, MA). The membrane was then blocked with 5% skim milk in Tween-20/PBS and incubated with primary antibodies: rabbit anti-ICAM-1 (1:1,000) and mouse anti-GAPDH (1:1,000). The blots were then incubated with HRP-conjugated secondary antibodies. The bands were visualized using the ECL system detection system (Pierce, Rockford, IL) and quantified with Image J software (NIH; Bethesda, MD) with a fixed rectangular box covering each band. The software automatically plots the lanes and provides an average intensity of pixels with a graphical peak. The band intensity was acquired after the peak area was closed off.

### Real-time polymerase chain reaction

Conﬂuent HUVECs in 6 cm plates were incubated with LPS (1 μg/ml) for 6 h in the presence or absence of GC31 (0.1–10 μM) or VP30 (10 μM). Total RNA was extracted with TRIzol reagent (Invitrogen, Grand Island, NY), according to the manufacturer’s instructions. Total RNA (2 μg) of each sample was reverse-transcribed into cDNA, and the ﬂuorescence quantitative real-time PCR (RT-PCR) was performed on the Rotor-Gene 3000 (Corbett Research, Mortlake, Australia) instrument with a One-Step SYBR PrimeScript RT-PCR kit (TaKaRa Bio Inc., Shiga, Japan) according to the protocol. Speciﬁc sense and antisense primers used were as follows: ICAM-1, sense: 5′-CAG TGA CCA TCT ACA GCT TTC CGG-3′, antisense: 5′-GCT GCT ACC ACA GTG ATG ATG ACA A-3′; GAPDH, sense: 5′-ACC ACA GTC CAT GCC ATC AC-3′; antisense: 5′-TCC ACC ACC CTG TTG CTG TA-3′. GAPDH was chosen as a housekeeping gene to compare the amount of total mRNA of each sample. The transcript number was calculated using a 2 −△CT method (relative) [20].

### Western blot analysis for nuclear factor kappa B and mitogen-activated protein kinase pathways

HUVECs were pretreated with GC31 (0.1–10 μM) or VP30 (10 μM) for 30 min and then incubated with LPS (1 μg/ml) for 30 min or 1 h. After treatment, cells were washed twice in PBS and rapidly harvested using a cell scraper. Nuclear and cytoplasmic extracts were prepared on ice with ProteoJET cytoplasmic and nuclear protein extraction kit (Fermentas Life Science, Opelstrasse, Germany). The whole cell lysates (40 μg) and nuclear extracts (30 μg) were separated with 10% SDS–PAGE and electrotransferred to PVDF membranes. After being blocked with 5% bovine serum albumin (BSA), the membranes were probed with primary antibodies, including IκBα, phospho-IκBα, p38, p-p38, ERK1/2, p-ERK1/2, or p-NFκB-p65 (Ser536). Each membrane was further incubated with HRP-conjugated goat antirabbit IgG (1:1000) secondary antibodies. Lamin A/C (1:1000) was chosen as a nuclear housekeeping protein in the nuclear extract. The bands were visualized using the ECL system detection system, and the band density was determined with Image J software.

### Statistical analysis

All experiments were repeated at least three times. The results were expressed as mean ± standard deviation (SD) and analyzed with one-way analysis of variance (ANOVA) followed by Bonferroni’s post hoc test using SPSS 16.0 (SPSS; Chicago, IL) software for multiple comparisons of mean values. p<0.05 was considered statistically signiﬁcant.

## Results

### Few effects of GC31 on the viability of human umbilical vein endothelial cells

**Figure 1:**
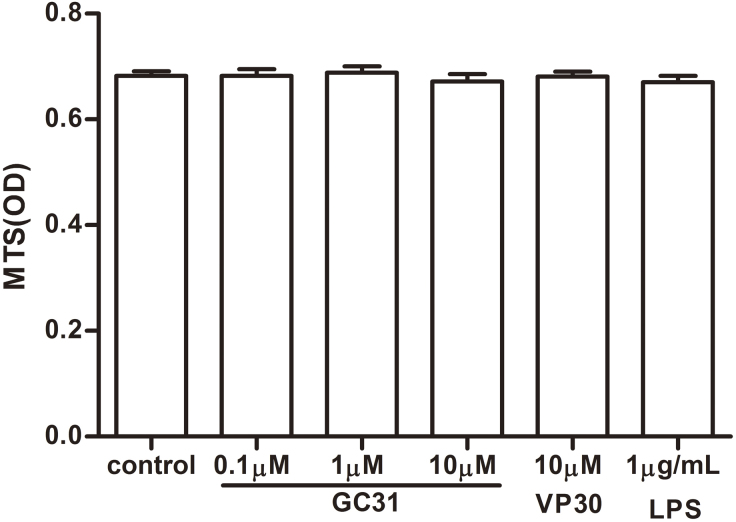
Few effects of GC31 on the cell viability of human umbilical vein endothelial cells. Confluent human umbilical vein endothelial cells (HUVECs) were incubated with GC31 (0.1, 1, and 10 μM), VP30 (10 μM), or LPS (1 μg/ml) for 24 h. The percentages of viable cells were measured using the CellTiter 96 aqueous one solution cell proliferation assay (MTS) kit. Results of the absorbance are the mean±SD (n=8).

To evaluate the effects of GC31, VP30, and LPS treatment on the growth of HUVECs, cell viability upon different concentration treatment was analyzed with MTS assay. Twenty-four hours post treatment with GC31 (0.1–10 μM), VP30 (10 μM), or LPS (1 μg/ml), compared to the control group treated with culture medium only, the absorbance of all treated groups showed no significant difference, suggesting GC31, VP30, and LPS have few inhibitory effects on HUVECs (p>0.05, Figure 1). Since MTS assay can also be used to evaluate cell proliferation, the insignificant toxic effect also indicated that the modulatory activities of peptides on HUVECs were irrelevant to inhibiting cell proliferation.

### GC31 treatment reduced U937 cell adhesion to lipopolysaccharide-induced human umbilical vein endothelial cells

**Figure 2:**
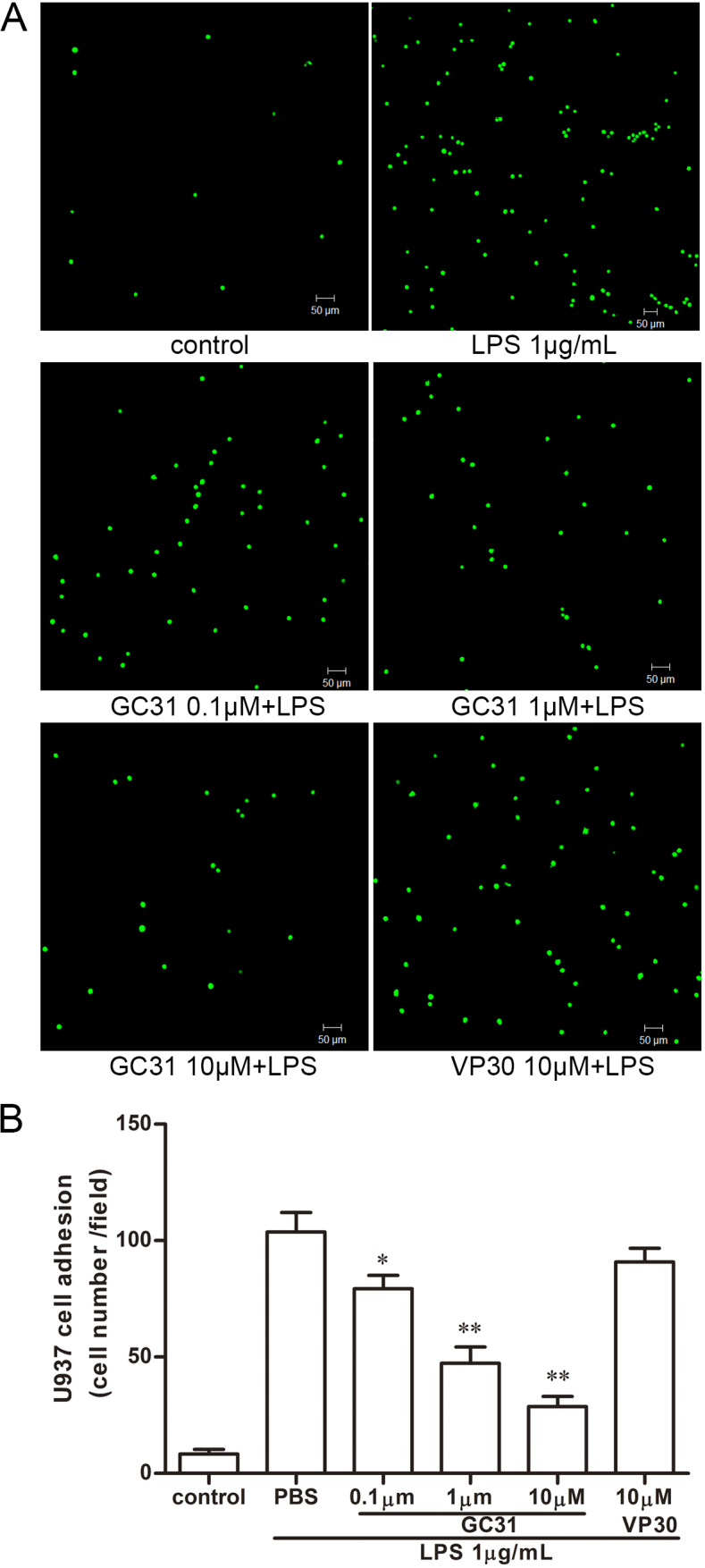
GC31 treatment reduced U937 cell adhesion to lipopolysaccharide-induced human umbilical vein endothelial cells. Human umbilical vein endothelial cells (HUVECs) were pretreated with GC31 (0.1, 1, and 10 μM), VP30 (10 μM), or vehicle for 18 h, exposed to lipopolysaccharide (LPS) (1 μg/ml) for 6 h, and then incubated with CM-H2DCFDA-labeled U937 cells for 30 min as described in Methods. **A**: Typical images of U937 cells adhered to HUVECs are shown. Green fluorescence represents adhered U937 cells. Scale bar=50 μM. **B**: Quantification of monocyte adhesion is presented by the number of adhered U937 cells per field counted under microscope. Data are expressed as means±SD (n=3). * p<0.05, ** p<0.01 compared to the LPS-treated group.

Our previous study showed that GC31 treatment could effectively prevent leukocyte infiltration in EIU [13] and it was demonstrated that the earliest infiltrated inflammatory cells into ocular tissue of EIU rats were mononuclear cells [21]. Accordingly, we next examined the adhesion of monocytes to LPS-activated HUVECs pretreated with GC31. The U937 cell line is a human cell line established from a diffuse histiocytic lymphoma and displaying many monocytic characteristics. It serves as an in vitro model to study the behavior and differentiation of monocytes and macrophages. With 1 μg/ml of LPS treatment alone for 6 h, the number of U937 cell adhesion to HUVECs was significantly increased 12.45-fold compared to the control group (p<0.01; Figure 2A,B). When HUVECs were pretreated with indicated concentrations of GC31 for 18 h before exposure to LPS, the number of adhered U937 cells was strikingly decreased in a dose-dependent manner (0.1 μM, 0.77 fold, p<0.05; 1 μM, 0.45 fold, p<0.01; 10 μM, 0.28 fold, p<0.01 compared with the LPS-treated group). Though VP30 (10 μM) pretreatment reduced the number of adhered U937 cells slightly, the difference was not statistically significant (Figure 2B, p>0.05).

### GC31 suppressed lipopolysaccharide-induced intercellular adhesion molecule-1 expression in human umbilical vein endothelial cells

**Figure 3:**
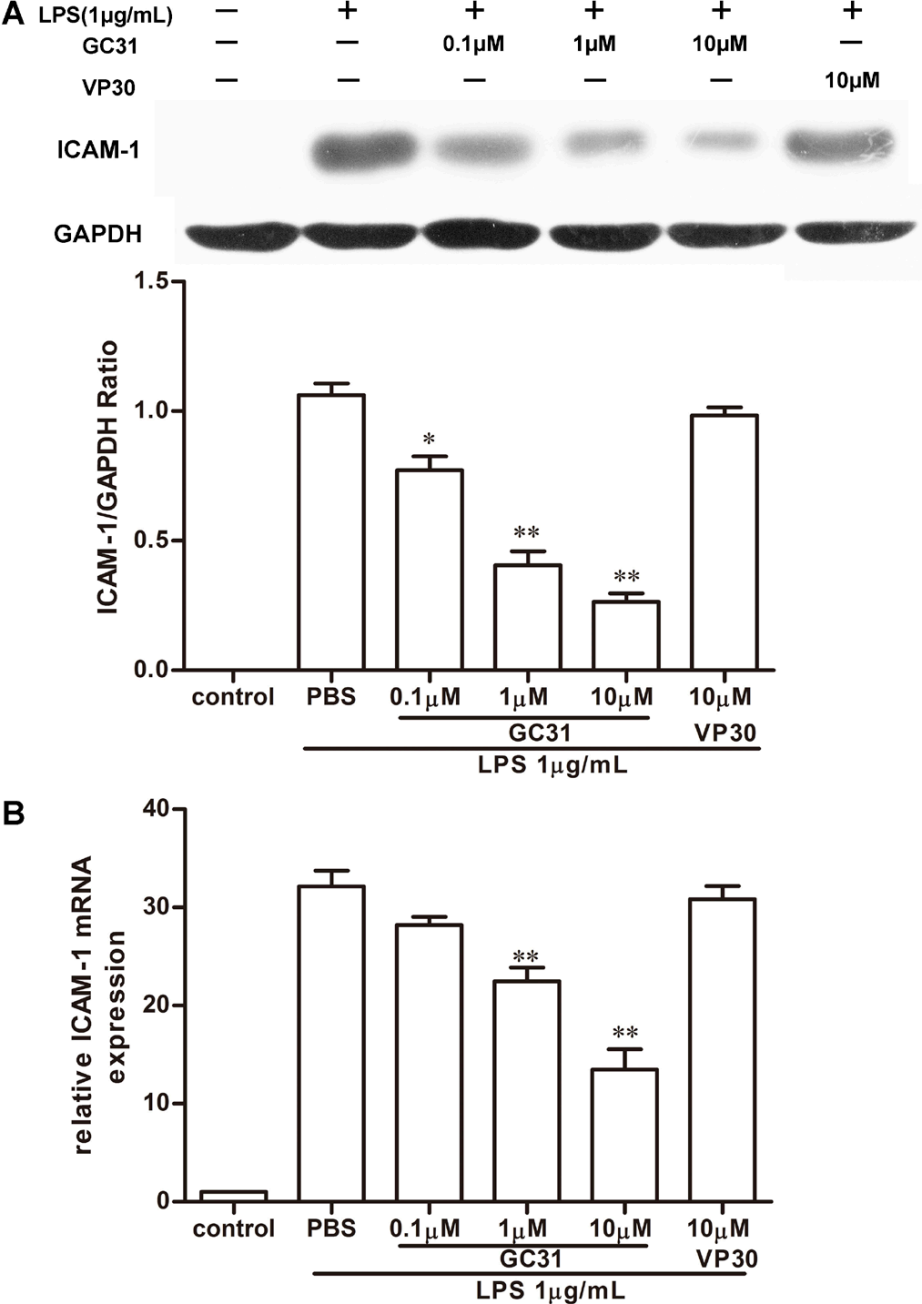
GC31 suppressed lipopolysaccharide-induced intercellular adhesion molecule-1 expression in human umbilical vein endothelial cells. The cells were treated with GC31 (0.1 μM, 1 μM, and 10 μM) or VP30 (10 μM) simultaneously combined with lipopolysaccharide (LPS) (1 μg/ml). **A**: The amounts of intercellular adhesion molecule-1 (ICAM-1) in the whole cell extracts 12 h after LPS stimulation were determined with western blot analysis. **B**: The messenger RNA (mRNA) levels of ICAM-1 were assessed with real-time PCR analysis 6 h after LPS stimulation. Glyceraldehyde-3-phosphate dehydrogenase (GAPDH) protein and mRNA were measured as the internal control. The data represent means±SD of triplicate measurements. *p<0.05, **p<0.01 versus the LPS group.

To investigate the mechanism in which GC31 decreased the binding of monocytes to HUVECs, we examined the expression of ICAM-1 in LPS-activated HUVECs, which is well known to mediate the firm binding of monocytes [9,22]. HUVECs were stimulated with 1 μg/ml of LPS in the presence or absence of GC31 or VP30 for 12 h. As indicated by the results of western blot analysis, exposure of cells to LPS (1 μg/ml) evoked a strong increase in ICAM-1 expression compared with unstimulated cells (Figure 3A). However, GC31 treatment resulted in a significant reduction in LPS-induced ICAM-1 expression in a dose-dependent manner, indicating that peptide GC31 was effective in blocking ICAM-1 expression in LPS-activated HUVECs. To further explore the effect of GC31 on modulating the transcriptional level of adhesion molecules, total cellular RNAs were isolated and analyzed with RT–PCR. Similar results were obtained that treatment with GC31 signiﬁcantly suppressed the induction of ICAM-1 mRNA in HUVECs stimulated with LPS for 6 h (Figure 3B). Altogether, these results suggested that the ICAM-1 expression induced by LPS is post-transcriptionally prevented by GC31.

### GC31 inhibited lipopolysaccharide-induced inhibitor of nuclear factor kappa B alpha degradation and nuclear factor kappa B p65 nuclear translocation

**Figure 4:**
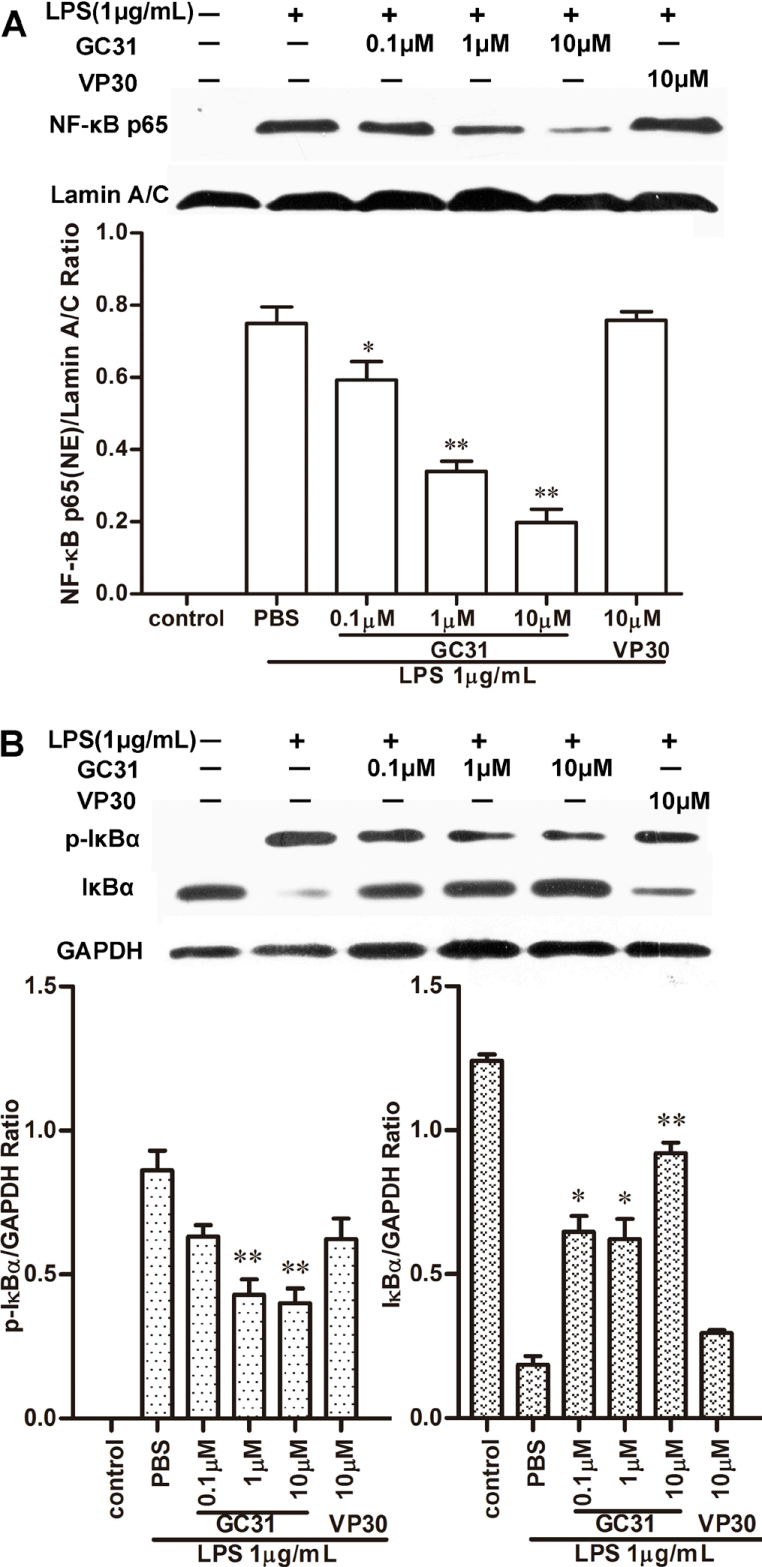
GC31 inhibited lipopolysaccharide-induced inhibitor of nuclear factor kappa B alpha degradation and nuclear factor kappa B nuclear translocation in human umbilical vein endothelial cells (HUVECs). **A**: Cells were preincubated with GC31 or VP30 for 30 min and then with 1 μg/mL lipopolysaccharide for 1 h. Nuclear extracts were analyzed with western blot analysis using antibody against phosphorylated nuclear factor kappa B (NF-κB) p65. Lamin A/C was used as the internal control. **B**: Human umbilical vein endothelial cells (HUVECs) were pretreated with GC31 (0.1–10 μM) or VP30 (10 μM) for 30 min, then stimulated with LPS (1 μg/ml) for 30 min, and the expression of inhibitor of nuclear factor kappa B alpha (IκBα) and phosphorylated IκBα (p-IκBα) in the protein extracts was determined with western blot. Glyceraldehyde-3-phosphate dehydrogenase (GAPDH) was used as the loading control. The band density was determined by Image J software and the results represent means±SD of three independent experiments. *p<0.05, **p<0.01 versus the LPS group.

NF-κB is a crucial transcription factor for expression of adhesion molecules [23,24]. The phosphorylation of NF-κB p65 (Ser536) plays an important role in regulating NF-κB activation and transcription of many inflammatory genes [24]. Therefore, we determined the effect of GC31 on NF-κB transcriptional activation. HUVECs were treated with various concentrations of GC31 for 30 min before stimulation with LPS for 1 h. Pretreatment with GC31 dose-dependently reduced the translocation of phosphorylated p65 NF-κB to the nuclear fraction compared to the LPS group (Figure 4A). Translocation of NF-κB from cytoplasm to the nucleus was preceded by the phosphorylation, ubiquitination, and proteolytic degradation of IκBα [25]. To explore whether GC31 affected LPS-induced degradation of IκBα, western blot analysis was performed. As Figure 4B shows, stimulation with LPS treatment for 30 min caused rapid phosphorylation and degradation of IκBα, while pretreatment with GC31 for 30 min inhibited the LPS-induced phosphorylation and degradation of IκBα in a dose-dependent fashion (Figure 4B). Taken together, the data suggested that GC31 inhibited LPS-induced NF-κB activation, which might be associated with the blockade of LPS-induced adhesion molecule production by GC31.

### GC31 reduced lipopolysaccharide-induced activation of mitogen-activated protein kinase pathways in human umbilical vein endothelial cells

**Figure 5:**
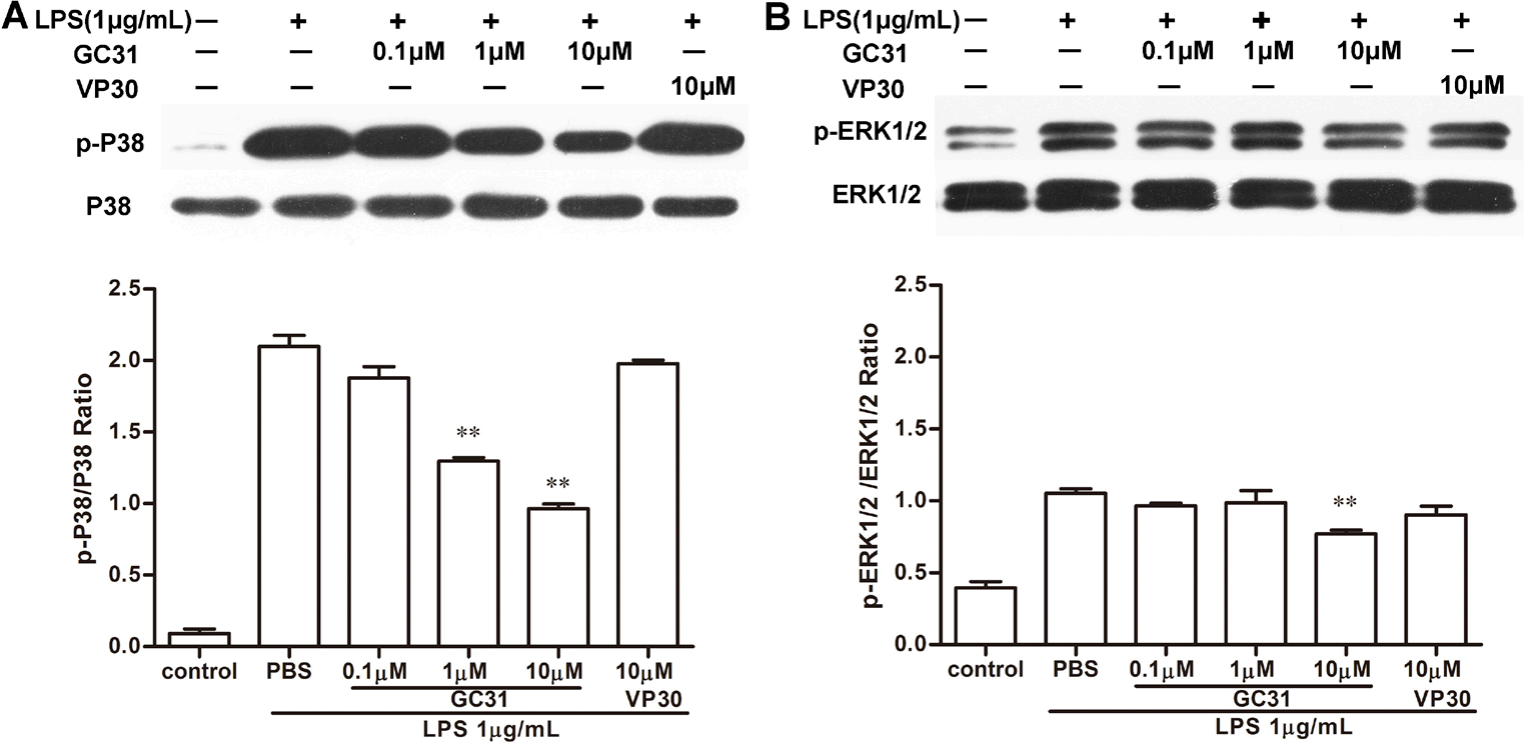
GC31 reduced lipopolysaccharide-induced activation of mitogen-activated protein kinase pathways in human umbilical vein endothelial cells. Human umbilical vein endothelial cells (HUVECs) were pretreated with or without indicated concentrations of GC31 for 30 min, followed by induction with 1 μg/mL lipopolysaccharide (LPS) for 30 min. The levels of phospho-p38 (**A**) and phosphoextracellular signal-regulated kinase-1/2 (p-ERK1/2) (**B**) were determined with western blot analysis. The relative levels were calculated as the ratio of the phosphorylated target protein to the total protein expression. Data were expressed as means±SD of three independent experiments. *p<0.05, **p<0.01 versus the LPS group.

Of the signaling pathways activated by LPS in endothelial cells, the p44/42 MAPK and p38 MAPK pathways have been shown to be directly involved in producing cytokines and adhesion molecules [26,27]. Hence, we examined the effect of GC31 on LPS-induced MAPK activation. As shown in Figure 5, 30 min after LPS treatment, the levels of activated p38 MAPK and ERK1/2 in the untreated cells were markedly increased. However, GC31 pretreatment moderately inhibited p38 MAPK activity in a dose-dependent manner and mildly reduced ERK1/2 activity in the LPS-stimulated HUVECs (Figure 5). These results suggested that GC31 may suppress LPS-induced ICAM-1 expression, at least in part, by reducing the activation of the p38 MAPK and ERK1/2 pathways.

## Discussion

Leukocyte-endothelial adhesion is an early step in many inﬂammatory disorders including sepsis [28], ischemia-reperfusion injury [29], atherosclerosis [30], and some ocular disorders including diabetic retinopathy [31,32] and uveitis [9,15]. The adhesion of monocytes to the endothelium is well established as a major early step in the development of atherosclerosis [33] as well as experimental uveitis [21]. In addition, it has been observed that preferential location of leukocyte adhesion occurs mainly in the endothelium of retinal veins and venules [14,16] while the sensitivity of arterial endothelium on LPS and tumor necrosis factor-α induction is much slower than that of venous endothelium [34]. In the present study, we investigated the effect of GC31 on monocyte U937-cell adhesion to LPS-activated HUVECs. We demonstrated that GC31 inhibited the elevated mRNA and protein production of ICAM-1 in LPS-induced HUVECs and reduced monocyte adhesion to LPS-activated HUVECs. The effects of GC31 were possibly due to its blockade of the NF-κB, p38 MAPK, and ERK1/2 signaling pathways.

ICAM-1 and leukocyte function-associated antigen-1 have been shown to play an important role in the pathogenesis of EIU and experimental autoimmune uveitis (EAU) [9,35]. It has also been reported that the expression of adhesion molecules increased progressively as EAU developed, with the increased ICAM-1 expression starting earlier and more closely associated with the sites of blood–retinal barrier breakdown [16]. In our present work, great overexpression of ICAM-1 was found in HUVECs treated with LPS (1 μg/mL). However, treatment with GC31 significantly and dose-dependently inhibited the mRNA and protein expression of ICAM-1. Meanwhile, similar results were obtained in the monocyte-endothelium adhesion assay. These results are in accordance with the observation that the recombinant lectin-like domain of TM can attenuate endothelial adhesion molecule expression and adhesion of polymorphonuclear leukocytes [17]. We have reported that GC31 peptide could reduce the level of monocyte chemoattractant protein (MCP)-1 in the aqueous humor of EIU rats [13]. MCP-1 is uniquely essential for monocyte recruitment and can direct migration of adherent cells across the endothelium [36,37]. Taken together, all the results suggested that GC31 has an important effect on preventing leukocyte-endothelium interaction under inflammatory conditions.

The architecture of ICAM-1 promoter contains a large number of binding sites for inducible transcription factors, including AP1, C/EBP, TFIID, Ets, NF-κB, etc [38]. The proximal NF-κB binding site located about 200 bp upstream of the translation start site has been shown to be particularly important for the induction of ICAM-1 transcription [39]. In resting cells, NF-κB exists in its canonical form as a p50/p65 heterodimer bound to the inhibitor factor-κB (IκB) in the cytoplasm. In response to extracellular stimulation such as LPS and tumor necrosis factor-α, the degradation of IκB through proteolysis is initiated, which allows the translocation of NF-κB from the cytoplasm into the nucleus [23,24]. Once translocated to the nucleus, p50/p65 binds to the κB sites in various genes, including elements in the E-selectin, vascular cell adhesion protein 1, and ICAM-1 promoters [23]. In the present study, GC31 pretreatment suppressed LPS-induced NF-κB activation by reducing the levels of NF-κB p65 in the nuclei of HUVECs and inhibiting the degeneration of IκBα in the cytoplasm, consistent with its effects on LPS-induced RAW264.7 cells [13]. Previous studies demonstrated that two different NF-κB inhibitors, PDTC and BAY11–7082, inhibited the expression of these adhesion molecules, thus preventing monocyte adhesion to endothelial cells [34,40]. Therefore, we speculate that the inhibitory effect of GC31 on ICAM-1 mRNA and protein production and monocyte-endothelium interaction in LPS-induced HUVECs may result from the suppression of NF-κB activation.

It has been reported that the MAPK family is involved in LPS-mediated signaling in endothelial cells and ICAM-1 expression [26,41-43]. Hence, we assessed the effect of GC31 on LPS-induced phosphorylation of p38 MAPK and ERK1/2. Our findings suggested that GC31 pretreatment mild to moderately suppressed the phosphorylation of both proinflammatory kinases in LPS-stimulated HUVECs, and the inhibitory effect on p38 MAPK activation is more prominent. In endothelial cells, p38 MAPK can mediate cellular responses through translocating to nucleus, where it phosphorylates and activates transcription factors, i.e., AP1 [44] and NF-κB [45]. Carter et al. [45] found that the p38 MAP kinase regulates NF-kB-dependent gene transcription, in part, by modulating activation of TATA-binding protein, and a dominant-negative p38 MAPK expression vector reduces NF-κB-dependent gene expression but has no impact on NF-κB activation at any levels. Thus, inhibition of p38 MAPK activation may further restrict other transcriptional factors’ effect, such as NF-κB, and then prevent the inflammatory signaling cascade.

In some ocular surface inflammatory diseases, such as dry eye and pterygium, ICAM-1 was found to be upregulated on lymphocytes and/or vascular endothelial cells resulting in lymphocytic diapedesis to the lacrimal and conjunctival tissues, and may serve as a signaling molecule for predisposition of ocular surface inflammation [46,47]. Because of its small size, the GC31 peptide may be promising for topical application. In addition, increased leukocyte adhesion and expression of proinflammatory cytokines and adhesion molecules have characterized several other ocular diseases, including diabetic retinopathy [48], retinal vessel occlusions, and ischemic-reperfusion injury [49]. The current findings about the novel peptide GC31 may be helpful in these conditions and deserves further investigation.

In conclusion, our results demonstrated that GC31 interfered with U937 monocyte adhesion to LPS-induced HUVECs by suppressing endothelial ICAM-1 mRNA and protein expression, through inhibiting the nucleus translocation of NF-κB, and partially by reducing the phosphorylation of p38 MAPK and ERK1/2. These findings underscore a role of GC31 in ameliorating endothelial dysfunction by exogenous insults and raise the possibility that GC31 plays a protective role in the pathophysiology of vascular inflammation and uveitis.
